# Chronic inflammation deteriorates structure and function of collagen fibril in rat temporomandibular joint disc

**DOI:** 10.1038/s41368-018-0036-8

**Published:** 2019-02-20

**Authors:** Sheng-Jie Cui, Yu Fu, Yan Liu, Xiao-Xing Kou, Jie-Ni Zhang, Ye-Hua Gan, Yan-Heng Zhou, Xue-Dong Wang

**Affiliations:** 1 Department of Orthodontics, Peking University School and Hospital of Stomatology, 22# Zhongguancun South Avenue, Haidian District Beijing, China; 2 Center for Craniofacial Stem Cell Research and Regeneration, Peking University School and Hospital of Stomatology, 22# Zhongguancun South Avenue, Haidian District Beijing, China; 3 Center for Temporomandibular Disorders and Orofacial Pain, Peking University School and Hospital of Stomatology, 22# Zhongguancun South Avenue, Haidian District Beijing, China

## Abstract

Collagen is the building component of temporomandibular joint (TMJ) discs and is often affected by inflammation in temporomandibular disorders. The macromechanical properties of collagen are deteriorated by chronic inflammation. However, the mechanism by which inflammation influences disc function remains unknown. The relationship between the ultrastructure and nanomechanical properties of collagen in inflamed discs should be clarified. Seven-week-old female Sprague–Dawley rats were randomly divided into two groups. Chronic TMJ inflammation was induced by intra-articular injection of complete Freund’s adjuvant, and samples were harvested after 5 weeks. Picrosirius staining revealed multiple colours under polarized light, which represented alternative collagen bundles in inflamed discs. Using atomic force microscopy scanning, the magnitude of Young’s modulus was reduced significantly accompanied with disordered collagen fibril arrangement with porous architecture of inflamed discs. Transmission electron microscopy scanning revealed a non-uniform distribution of collagen fibres, and oversized collagen fibrils were observed in inflamed discs. Fourier transform infrared microspectroscopy revealed a decrease in 1 338 cm^−1^/amide II area ratio of collagen in different regions. The peak positions of amide I and amide II bands were altered in inflamed discs, indicating collagen unfolding. Our results suggest that sustained inflammation deteriorates collagen structures, resulting in the deterioration of the ultrastructure and nanomechanical properties of rat TMJ discs.

## Jaw pain: Unraveling the causes

Chronic inflammation damages the collagen disk that cushions the temporomandibular joint (TMJ) of the jaw, contributing to jaw disorders. Although chronic inflammation is known to harm collagen, its effects on TMJ disks were unclear. Xue-Dong Wang and Yan-Heng Zhou and coworkers at the Peking University School and Hospital of Stomatology in Beijing used advanced imaging technologies to investigate how inflammation affects TMJ collagen in rats. In TMJ disks, collagen is composed of long, intertwined threads called fibrils. Inflammation damaged the collagen’s chemical structure, preventing correct winding, and resulting in oversized and unevenly arranged fibrils. The TMJ disks were overly porous, with poor elasticity. The researchers hypothesize that these changes thicken and deform TMJ disks, leading to disk displacement and jaw disorders. Further studies are needed to determine whether anti-inflammatory treatments can protect TMJ collagen.

## Introduction

Temporomandibular disorders (TMDs) are a large family of diseases affecting the structure and function of the stomatognathic system.^1^ The temporomandibular joint (TMJ) disc absorbs peak stress during mandibular movement; thus, the disc effectively protects the TMJ.^2^ Abnormal temporomandibular disc function plays a significant role in pathogenesis of TMD. Disc displacement is one of the most common subtypes of TMD; however, its pathologic mechanism is unclear.

A normal disc has a concave configuration, which is consistent with the surface of condyle and temporal bone. Most studies have reported that disc deformation contributes to disc displacement and is often accompanied by inflammation and pain.^3,4^ However, different opinions exist. In several cases, TMJs exhibit inflammation and disc degeneration without disc displacement.^5,6^ Early studies on cadavers revealed that the deformation of the TMJ disc is associated with synovial inflammation without displacement; previous animal studies have reported that induced chronic inflammation of TMJ results in disc degeneration, including thickening and deformation.^7^ In addition, chronic inflammation of the TMJ could lead to the deterioration of macroscopic mechanical properties of discs, including a decrease in the tensile and compression modulus.^8^ Therefore, our group proposes an innovative theory: “inflammation may be the key factor leading to TMJ disc displacement”.

Stress distribution in the TMJ disc during mandible movement exhibits significant differences in normal and disc displacement groups;^9^ a disc with displacement endures more stress.^10^ Several findings have revealed the close relationship between disc structure and function.^11^ Studies have indicated that the arrangement of collagen fibrils influence tensile ability, and glycosaminoglycan influences compressive ability.^12^ However, how disc structural alteration results in biomechanical changes remains unclear. Moreover, little information is available regarding the relationship between inflammation and collagen deterioration in TMJ discs.

Complete Freund’s adjuvant (CFA) has been widely used for inducing acute or chronic TMJ inflammation by intra-articular injection.^7,8,13,14^ In our previous study, we found that the thickness, matrix content, biomechanical properties and surface structure of rat TMJ discs presented remarkable changes after CFA-induced chronic TMJ inflammation, and these changes result in the exposure and degradation of sub-superficial collagen fibrils.^7,8^ These results suggested that chronic inflammation in the TMJ could lead to the deterioration of mechanical properties and alteration of the disc surface ultrastructure, which might contribute to TMJ disc displacement. These previous studies prompted us to evaluate the pathologic alteration of internal collagen fibres to clarify the process of collagen degradation under sustained-inflammation conditions.

In the present study, we hypothesize that chronic inflammation contributes to the deterioration of the nanomechanical properties and ultrastructure of collagen fibres in TMJ discs. Considering that synovitis and internal derangement exhibit increased prevalence in females compared with males, we chose female rats to induce chronic TMJ inflammation similar to our previous studies. We applied picrosirius red staining, atomic force microscopy (AFM), transmission electron microscopy (TEM), Fourier-transformed infrared microspectroscopy (FTIRM) and circular dichroism to evaluate the changes in the arrangement, nanomechanical properties, ultrastructure and chemical structure of collagen fibrils in inflamed TMJ discs. An explanation of the mechanism of inflammation-induced degeneration of TMJ discs from the view of collagen fibres was provided.

## Results

### Arrangement of different collagen fibres in picrosirius red staining

**Fig. 1:**
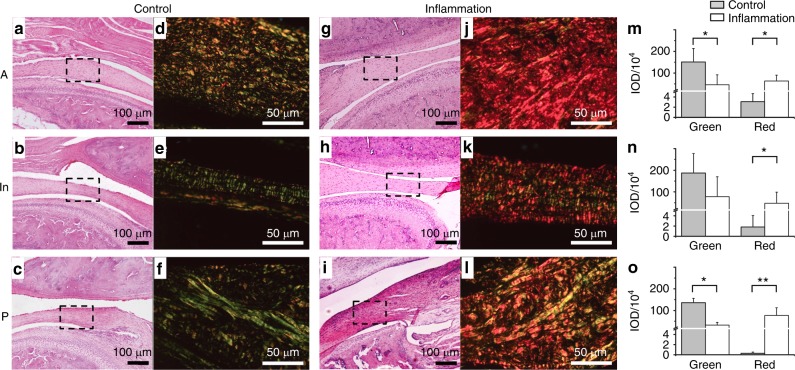
Histopathologic changes of TMJ discs. **a**–**c** Representative microscopic features of the discs from the control group subject to HE staining. The disc was thinner in the intermediate zone and thicker in the anterior and posterior bands. **d**–**f** Representative images of the disc from the control group assessed by picrosirius staining under polarized light. Collagen bundles presented yellowish to green reflection, and distribution of different colours was organized. The black dotted squares in **a**–**c** are magnified in **d**–**f**, respectively. **g**–**i** Representative microscopic features of the discs from the inflamed group subject to HE staining. The disc was thickened in all three regions. **j**–**l** Representative image of the disc from the inflamed group assessed by picrosirius staining under polarized light. Collagen bundles exhibited varied colours, but yellow to pink dominated. The distribution of different colours was disordered. The black dotted squares in **g**–**i** are magnified in **j**–**l**, respectively. **m**–**o** Inflammation decreased green intensity and increased red intensity in anterior and posterior bands significantly and exhibited the same tendency noted in the intermediated zone without significant differences. *Y*-axis represents IOD (integrated optimal density of positive pixel area). A, anterior band; In, intermediate zone; P, posterior band. *P *<* 0.05, **P *<* 0.01

Picrosirius red staining is proportional to the content and orientation of collagen fibre in the extracellular matrix of TMJ discs^15,16^ and significantly facilitates the detection of collagen bundle arrangements. The inflamed and control discs exhibited considerable differences during polarizing microscopy. Haematoxylin and eosin (HE) staining for routine microscopy revealed the distinct locations of TMJ discs. Picrosirius red staining revealed the detailed morphology of the collagen fibres of discs at high magnification (Fig. 1). Although the orientation of collagen fibres was heterogeneous in the control group, the fibres were well organized. Compared with the control group, inflamed collagen fibres were disordered. The collagen fibres from the control group appeared greenish-yellow to yellow in most positions of the discs in the anterior band, intermediate zone and posterior band. The inflamed discs exhibited apparent red and yellow refraction when observed under polarized light. In the anterior band, the collagen fibres in the entire area exhibited a predominant red colour, and the green colour significantly diminished. In the intermediate zone and posterior band, the collagen fibres exhibited multiple colours, including yellow, orange and red. The greenish-yellow to red colour under polarized light indicates the changes of collagen arrangement and type as well as the transition from fine fibres to thick collagen fibre bundles.^17^ The statistical analysis of integrated optical density showed the significant differences of the two groups in the anterior and posterior bands. In addition, it showed the same tendency in the intermediate zone without significant differences of greenish colour.

### Micro-morphology is disordered in inflamed discs by AFM imaging

**Fig. 2:**
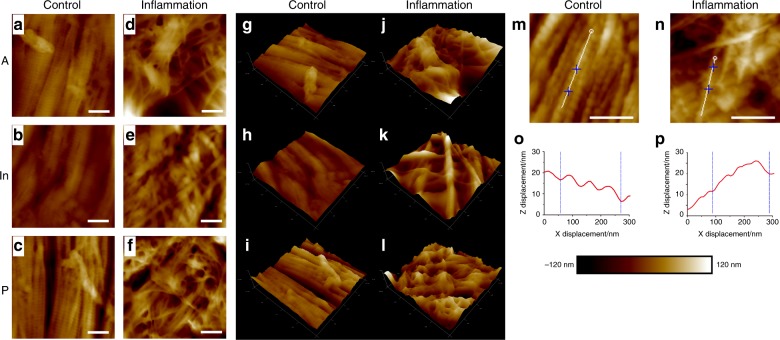
AFM mapping scanning images of TMJ discs. **a**–**c** Representative morphologic features of discs from the control group. The collagen fibrils exhibited good arrangement with clear light and dark banding of the D-period. The arrangement of anterior bands and intermediate zones were similar, and the posterior band was slightly loosened but still in good order. **d**–**f** Representative morphologic features of the discs from the inflamed group. The banding of collagen fibrils was diminished, and many single fibrils were observed. The spatial arrangement of collagen bundles was disordered and non-directional. **g**–**l** Three-dimensional reconstruction of **a**–**f** images, respectively. The inflammation group presented porous and undulated structures compared with the control group. Representative images of collagen single micro-fibrils from control and inflamed TMJ discs, respectively. **o**, **p** *x*–*z* directional curve of single collagen micro-fibril corresponding to the white line in **m** and **n**. The topographic structure exhibited good repeating D-periods in control discs and degraded performance in inflamed discs. Scale bar: 400 nm

Representative mapping images from various locations of the control and inflamed discs are presented in Fig. 2a–l. The arrangement of the collagen micro-fibrils in the control discs was regular and directional. The bundles consisted of distinctive collagen micro-fibrils, and the clearance between adjacent bundles was narrow in the anterior band and intermediate zone. In the posterior band, the clearance of collagen bundles was wider compared with the other two locations. Although the control samples exhibited various morphologies, tropocollagen molecules staggered from each other by approximately 67 nm, which is called D-period,^18^ exhibited a good performance in all of the mapping images from different positions. Both the arrangement and structure of collagen micro-fibrils in the inflamed samples were disordered and exhibited large interspace between adjacent bundles. The magnitude of irregular collagen bundles increased after induced inflammation.

The internal morphological changes in the collagen fibrils were analysed using AFM. The ultrastructure of collagen micro-fibrils is presented in Fig. 2m–p. In the control group, collagen micro-fibrils presented a clear D-period, and the repeated cycle was approximately 67 nm, which represented typical collagen ultrastructure. However, in the inflammation group, the inflamed collagen micro-fibrils exhibited an attenuated D-period and disordered ultrastructure.

### Collagen fibrils became hypertrophic in inflamed discs as assessed by TEM scanning

**Fig. 3:**
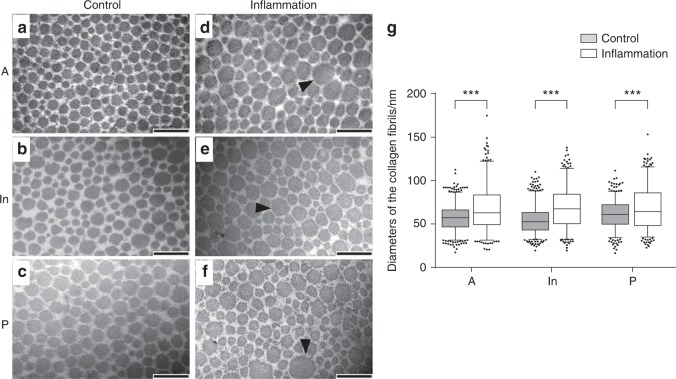
TEM analysis of internal section of discs of TMJs with or without inflammation. **a**–**c** Representative cross-sections of discs from the control group. Collagen fibril diameter ranged from 20 to 120 nm with 40–70 nm noted most often. **d**–**f** Representative cross-sections of the discs from the inflamed group. Some large collagen fibrils (*d* > 120 nm) were observed, and the distribution was heterogeneous. Black arrows indicate abnormal hypertrophic collagen fibrils. **g** Nonparametric tests of collagen fibril diameter distributions based on three rats per group. The box presents 5%, lower quartile, median, higher quartile and 95%. The black dots represent fibrils count <5% or >95%. Large collagen fibrils appeared after inflammation was induced. The control and inflammation groups exhibited significant differences in collagen diameter. ***P *<* 0.001, scale bar: 200 nm

The collagen fibres in the control group were configured regularly with similar diameters and spaces. However, in the inflamed discs, the distribution of the diameters of collagen fibres manifested a broad range, and hypertrophy of collagen fibrils was observed in all the three bands. The oversized collagen fibrils, especially fibrils with a diameter of >120 nm, dramatically increased in most areas of the entire disc (Fig. 3). The distribution of the diameters of collagen fibrils revealed significant differences between the control and inflammation groups (*P* *<* 0.001) in all the three bands.

### Elastic modulus of collagen decreased in inflamed discs as assessed by the AFM test

**Fig. 4:**
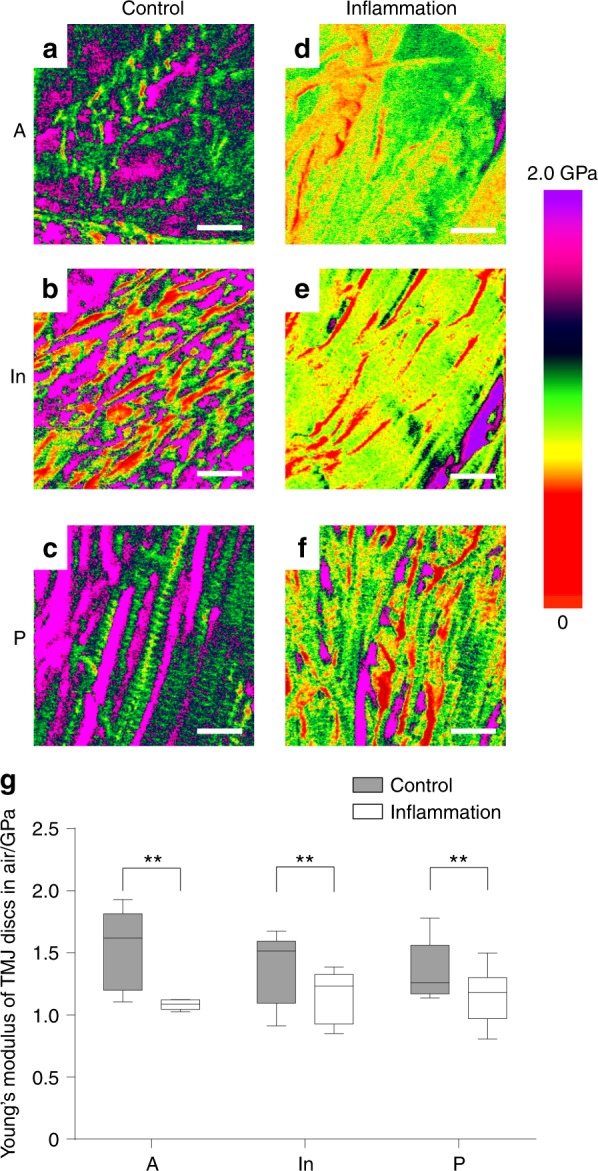
AFM mechanical scanning images of TMJ discs. **a**–**c** Representative nanomechanical mapping features of discs from the control group. Magnitude of Young’s modulus was consistent with the collagen D-period banding. **d**–**f** Representative nanomechanical mapping features of discs from the inflamed group. Compared with the control group, the inflamed samples exhibited a decreased magnitude and disordered distribution of Young’s modulus. **g** Nonparametric tests of collagen fibril diameter distribution based on three rats per group. The box presents 5%, lower quartile, median, higher quartile and 95%. The inflammation group exhibited significant differences in Young’s modulus compared with the control group. **P *<* 0.01, scale bar: 400 nm

The Derjaguin–Muller–Toporov model was employed to calculate elastic modulus during AFM scanning.^19^ Representative colour-coded mapping images revealed a visible difference between the control and inflamed groups. The elastic modulus of a different location exhibited a small range in the control group (anterior band: 1.0–2.0 GPa, median: 1.6 GPa; intermediate zone: 0.8–1.9 GPa, median: 1.5 GPa; posterior band: 0.8–1.9 GPa, median: 1.3 GPa). Compared with the control group, the magnitude of the elastic modulus decreased significantly in the anterior band (0.9–1.3 GPa, median: 1.1 GPa), intermediate zone (0.6–1.7 GPa, median: 1.2 GPa) and posterior band (0.6–1.6 GPa, median: 1.2 GPa) in the group with inflamed discs. The reduction in the modulus represents the capacity to resist extrinsic stress, which results in the deterioration of the nanomechanical property of collagen fibrils (Fig. 4).

### Integrity damage detected through FTIRM and circular dichroism under inflammation conditions

**Fig. 5:**
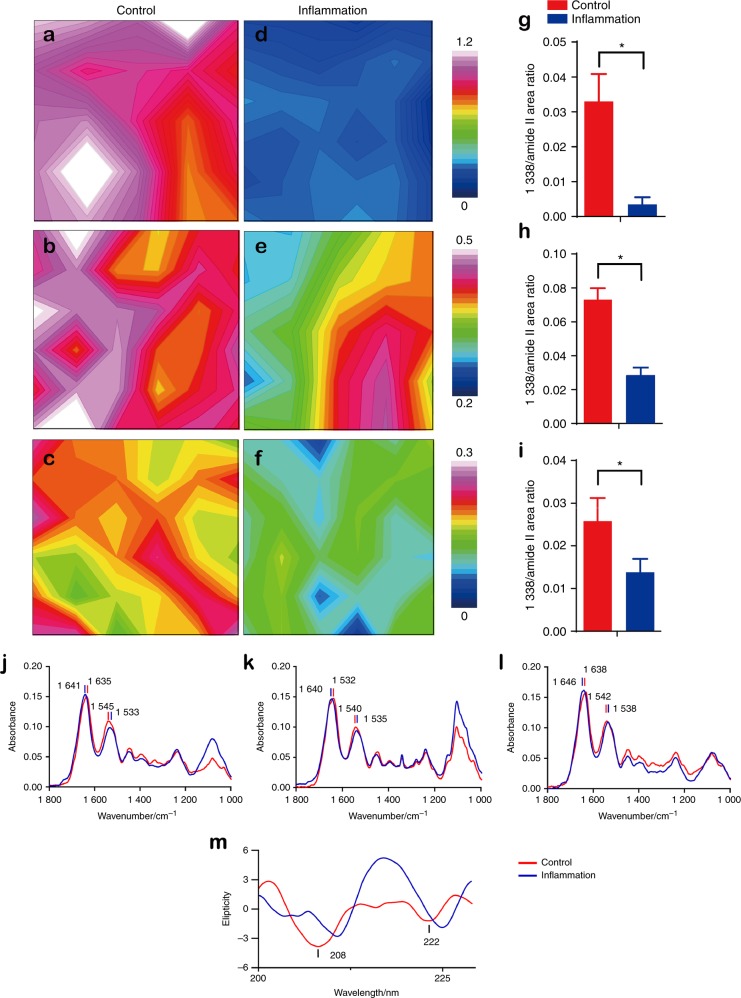
FTIRM mapping images of TMJ discs. **a**–**f** Mapping images of the integration area of 1 338 cm^−^^1^. The magnitude of the inflammation group was decreased compared with the control group, and the distribution was diverse in inflamed discs. **g**–**i** The 1 338 cm^−1^/amide II area ratio exhibited a significant decrease in the inflammation group in the anterior band, intermediate zone and posterior band, respectively. **j**–**l** Representative single spectra of the TMJ discs (from the anterior band). In the anterior band, intermediate zone and posterior band, the amide I peak position moved to a large wave-number, and the amide II peak position moved to a small wave-number. **m** Circular dichroism of the TMJ disc collagen solution. The negative absorbances of 208 and 222 nm were distinctive in the control group, but less pronounced in the inflammation group

FTIRM represented the spectra of the chemical group of collagen in the control and inflamed discs. Collagen absorbance at 1 338 cm^−1^ is sensitive to a decrease in intensity when collagen degrades.^20^ The colour-coded mapping images present the different distributions and magnitudes of the 1 338 cm^−1^ peak area. The peak area decreased significantly in the inflamed TMJ discs (Fig. 5a–i). The comparison of spectral parameters and the 1 338 cm^−1^/amide II (1 483–1 585 cm^−1^) area ratio between the control and inflamed specimens revealed the differences between normal and inflamed TMJ discs.^21^ After the induction of chronic inflammation, the 1 338 cm^−1^/amide II area ratio decreased significantly in the anterior, intermediate and posterior bands. The representative spectra from all the FTIRM results are presented in Fig. 5j–l. Alterations in amide I in the posterior band (from 1 638 to 1 646 cm^−^^1^) and amide II in the anterior band (from 1 545 to 1 533 cm^−1^) were apparent and revealed deterioration of the primary collagen structure. Circular dichroism revealed alterations in the secondary structure of collagen as demonstrated by negative peaks at 208 and 222 nm in the control group and characterized by an α-helix.^22^ However, the peaks diminished in the inflamed sample (Fig. 5m), reflecting uncoiling of the α-helix.

## Discussion

The aim of the present study was to elucidate possible mechanisms of sustained inflammation resulting in degenerative changes in TMJ discs. As one of the main components of TMJ discs, collagen contributes to disc structure and function.^11^ In the present study, we discovered structural disorganization in inflamed collagen fibres through histological staining. Then, we detected the ultrastructure of collagen fibrils by TEM and AFM scanning. The results revealed that collagen fibril dimensions in inflamed discs were disordered and characterized by increasing hypertrophic and small fibrils. In addition, ultrastructure disorganization of collagen micro-fibrils and degradation of collagen single fibril were also typical in inflamed discs. Simultaneously, the elastic modulus of collagen fibrils decreased significantly in inflamed discs. To elaborate the mechanism involved in the changes in the ultrastructure and nanomechanical properties of inflamed discs, we analysed primary and secondary collagen structures using FTIRM and circular dichroism. We observed alterations in the primary and secondary structures based on absorbance at 1 338 cm^−1^ and amides I and II, and collagen presented uncoiling during inflammation. We suggest that inflammation-induced collagen molecule structure alternations contribute to deterioration of collagen ultrastructure, further influence nanomechanical properties and finally result in TMJ disc dysfunction.

The results of structural testing demonstrated how inflammation deteriorates the collagen structure of TMJ discs. Picrosirius staining demonstrated the derangement and change in collagen fibre levels between the control and inflamed groups. AFM scanning revealed that the deterioration of collagen nanofibrils may result in histological changes in collagen bundles. In consideration of the partial disappearance of the staggered banding of collagen fibrils, AFM scanning images imply the disruption of the intermolecular connections in collagen nanofibrils. Given that the collagen fibrils were composed of tropocollagens connected to one another by covalent and hydrogen bonds,^23^ sustained inflammation may deteriorate the original tropocollagen structure. Once the collagen structure was imperfect, the D-period and nanomechanical indentation modulus exhibited significant reductions.^24^ The three-dimensional (3D) reconstruction results clearly revealed the disordered arrangement of inflamed collagen fibrils in 3D space. Collagen fibrils in adult rats varied widely in diameter (range: 10–120 nm; peak: 40–70 nm). TEM revealed that the inflamed discs exhibited significant non-uniformity in collagen micro-fibril dimensions that manifested as abnormal thick fibrils (*d* > 120 nm). The diameters and the arrangement of control collagen fibrils exhibited pre-dominant middle-diameter fibrils with small-diameter fibrils wedged between the middle-diameter fibrils. This pattern matches the function of enduring mechanical stress.^25^ However, in inflamed discs, the enlargement of collagen fibrils, especially hypertrophy, disturbed the regular alignment of collagen fibrils. This feature may contribute to reductions in the nanomechanical property of collagen in the inflamed discs. This enlargement of collagen fibrils was similar to the age-related changes in rat TMJ discs^26^ However, compared with the physiological ageing process, inflammatory degenerative alterations were attributed to the disordered repair of inflamed collagen.

AFM has been applied increasingly in recent years to detect the nanomechanical and ultrastructural changes in cartilage and fibrous tissues with inflammation.^27–30^ To the best of our knowledge, this study is the first to test the ultrastructural and nanomechanical properties of TMJ discs using AFM. The elastic moduli of the inflamed discs decreased, which is consistent with our previous macroscopic result demonstrating that the instantaneous Young’s moduli of the inflamed discs decreased significantly compared with the control group.^8^ Given that the microarchitecture of collagen fibrils was destroyed simultaneously by sustained inflammation, we believe that the internal changes resulted in the deterioration of the mechanical properties of TMJ discs. Nevertheless, as AFM mapping could not separate single fibrils from the sample, further studies are needed to determine whether the elastic modulus of single collagen was influenced by inflammation.

FTIRM and circular dichroism analysis revealed the structural deterioration of inflamed collagen proteins. Several indexes can be used to evaluate collagen degradation by FTIRM; these indexes have been widely utilized to observe early cartilage degeneration in osteoarthritis samples.^31,32^ The absorbance at 1 338 cm^−1^, which represents collagen CH_2_ side chain vibrations, decreased when collagen is degraded.^21^ The changes in 1 338 cm^−1^ normalized to the amide II area ratio in different regions represents collagen damage.^33^ The decreased absorbance at 1 338 cm^−1^ indicates a change in collagen fibril structure and reveals the loosening bond between collagen molecules. This loosening may affect the biomechanical properties of collagen. Primary molecular vibrations related to these wave-number absorbances include amide I carbonyl stretching (C = O), amide II out of phase in-plane N–H deformation and C–N stretching.^34^ The shift of the amide I and amide II peak location was related to intramolecular and intermolecular hydrogen deterioration, and the secondary α-helix structure was also decreased. Sustained inflammation upregulated the expression of matrix metalloproteinases (MMPs),^35^ which interact with collagen peptide. These changes result in collagen unfolding, destroyed the hydrogen bond of the active site and formed the looped-out collagen chain.^36^ These protein structural changes partly explain why the collagen fibrils exhibited hypertrophy and a disordered arrangement. The collagen fibril structure is responsible for the elastic viscosity of TMJ discs.^11,37^ Although the structure of collagen fibrils in different regions is anisotropic,^38^ all three regions exhibited the same changing tendency of the elastic modulus in the present study. Deterioration of biomechanical properties possibly resulted from collagen degradation.^39^ Furthermore, collagen degradation formed a porous structure between collagen bundles that caused the elastic modulus to decrease in the entire region of inflamed discs.

Inflammation could also change the micro-environment of extracellular matrix, which directly influences collagen synthesis and assembly. Our previous study revealed that under the condition of chronic inflammation, the total content of collagen was dramatically increased,^7^ which indicates the “swelling” of inflamed disc ascribed to abnormal collagen synthesis. After procollagen is secreted out of the disc cell, an aberrant micro-environment may result in disturbed collagen fibril structure. Additionally, proteinases, such as a disintegrin and metalloproteinase with thrombospondin motif 2 (ADAMTS2) and MMPs, are involved in the process of collagen fibril maturing and assembly.^40,41^ Thus, both the synthesis and degradation of collagen fibrils were influenced during the sustained inflammation, which could explain the possible mechanism involved in the deterioration of collagen structure and function.

However, further studies are required to elucidate the intensive connection between structure and function and determine the possible signal pathway involved during the process of collagen metabolism in the inflammatory micro-environment. Intensive studies are needed to elucidate whether the changes in the structure and mechanical properties of collagen fibrils could be prevented by anti-inflammation treatment and whether disc displacement in clinical TMD patients can be induced by chronic inflammation in an animal model.

In conclusion, sustained inflammation influences collagen structures and results in the deterioration of the ultrastructure and nanomechanical properties of TMJ discs, which may change the stress distribution during mandible movement and could represent a key factor in disc displacement.

## Methods

### Induction of inflammation

Seven-week-old female Sprague–Dawley rats (180–200 g) were randomly divided into inflammation and control groups. TMJ inflammation was induced by two intra-articular injections of CFA (Sigma) for 5 weeks as previously described.^7^ The inflammation group was injected with 25 μL CFA (emulsified in 25 μL saline) in the upper compartment of the TMJs on days 0 and 14. The control group was injected with equivalent saline.

All rats were sacrificed by pentobarbital overdose on day 35. The animal procedures were approved by the Peking University Animal Ethics Committee prior to the initiation of the study (Approval number: LA2014221). The methods employed were performed in accordance with approved guidelines.

### Tissue preparation

For histological staining, entire TMJs were removed from three rats in each group, fixed in 4% paraformaldehyde in 0.1 mol•L^−1^ phosphate-buffered saline, and demineralized in 15% ethylenediaminetetraacetic acid. The specimens were dehydrated in graded alcohol and xylene, embedded in paraffin, and sectioned to 4 µm thickness (*n* *=* 3).

For AFM and FTIRM, we harvested TMJ discs from three rats per group. Each disc was cut into anterior, intermediated, and posterior segments; embedded by Tissue-Tek (Sakura Finetek, Torrance, CA, USA); and stored at −80 °C for cryopreservation.^42^ The specimens were sectioned to 40 µm thickness parallel to the disc surface using a cryostatic microtome at −40 °C.^27^ All the sections were stored at −40 °C before testing.

### HE and picrosirius red staining

The tissue sections were stained with HE to locate the different segments of discs. The sections adjacent to the HE staining samples were subjected to picrosirius red staining by incubation in sirius red (0.1% in saturated picric acid; #43 665, Fluka) for 1 h at room temperature. Subsequently, the sections were examined through polarizing microscopy. The green and red colours of the polarized images were analysed using Image-Pro Plus 6.0 according to the positive pixel per area (*n* *=* 3). Integrated optimal density of green and red colour was calculated in every image. To avoid subjective factors causing errors, the investigator was blind to the samples and groups.

### AFM imaging

Imaging was performed using an Icon atomic force microscope as previously described.^43^ The results of AFM imaging and nanoindentation obtained from three rats were analysed with NanoScope Analysis 1.40. The samples were imaged in Peak-Force QNM mode.^18^ A TAP525A silicon cantilever was utilized, and deflection sensitivity was calibrated using a sapphire model. Calibration of the spring constant and tip radius was conducted using a standard model with a known elastic modulus of 2.7 GPa as a reference; the value of the spring constant was 2.5–3 N•m^−1^, and the value of the tip radius was 12–15 nm.

To calculate the elastic modulus, three scans of representative locations were performed for each specimen. Each scan generated a mapping image with 256 × 256 resolution for the elastic modulus. Ten 100 nm × 100 nm samples were randomly selected from the collagen fibril region in each mapping image.

### TEM analysis

Three TMJ discs per group were fixed with 2.5% glutaraldehyde solution (Sigma) at 4 °C for 12 h, and TEM was performed as described previously.^44^ The discs were double-fixed with 1% osmium tetroxide (Sigma) and stained with lead citrate and uranyl acetate. Then, the anterior band, posterior band and intermediate zone of the discs were embedded in epoxy resin. Transverse ultrathin sections (100 nm) were obtained vertical to the disc surface. Counting and measurement of the diameter of collagen from the TEM images were accomplished by marking the fibrils individually in Image-Pro Plus 6.0 software. Collagen fibrils with diameters >120 nm were regarded as irregular.

### FTIRM and circular dichroism testing for chemical changes in collagen

The contiguous sections of the AFM specimens were assessed using FTIRM. The chemical changes in areas of the anterior band, intermediate zone and posterior band were examined. To obtain the requisite spatial resolution, measurements were performed on the infrared beam line in the laboratory of Bruker Corporation (Beijing, China). Interferograms were simultaneously collected from each element of the 25 points (5 × 5 lattice). LUMOS stand-alone micro-FTIR (Bruker, USA) was employed at a resolution of 1 cm^−1^ with 32 s per point. The imaged sample size was 60 µm × 60 µm. To obtain the spatial distribution of collagen amount, the results of absorbance at 1 338 cm^−^^1^ were plotted as colour-coded mapping images. Furthermore, collagen structure damage ratios (1 338 cm^−^^1^/amide II) from 15 infrared spectra were calculated to assess the inflamed damage of TMJ discs in distinct locations. FTIRM spectra were inputted and analysed using the OPUS 6.5 software (Bruker, USA). Absorbance peaks from individual spectrum were automatically recognized by the software, and the location of the amide I and amide II peaks were recorded.

For circular dichroism testing, the TMJ discs were immediately stored in liquid nitrogen after dissection and then dissolved in deionized water with ultrasonic oscillation for 30 min. Circular dichroism spectra were recorded between 190 and 300 nm with a J-810 spectropolarimeter (Jasco, Japan) at 20 °C.

### Statistical analysis

Statistical analysis was performed using the SPSS 11.0 software. The nonparametric Kolmogorov–Smirnov test was implemented to compare the differences in collagen diameter and elastic modulus between the control and inflammation groups. Statistical comparisons of the picrosirius red staining and FTIRM results were performed using independent *t* tests. *P* *<* 0.05 was considered significant.

### Data availability

The authors declare that the data supporting the findings of this study are available within the paper.

## References

[1] Peck CC (2014). Expanding the taxonomy of the diagnostic criteria for temporomandibular disorders. J. Oral Rehabil..

[2] Tanaka E (2003). Dynamic compressive properties of porcine temporomandibular joint disc. Eur. J. Oral Sci..

[3] Roh HS, Kim W, Kim YK, Lee JY (2012). Relationships between disk displacement, joint effusion, and degenerative changes of the TMJ in TMD patients based on MRI findings. J. Craniomaxillofac. Surg..

[4] Uehara J (2004). Soluble tumour necrosis factor receptors in synovial fluids from temporomandibular joints with painful anterior disc displacement without reduction and osteoarthritis. Arch. Oral Biol..

[5] Kondoh T, Westesson PL, Takahashi T, Seto K (1998). Prevalence of morphological changes in the surfaces of the temporomandibular joint disc associated with internal derangement. J. Oral Maxillofac. Surg..

[6] de Bont LG, Boering G, Liem RS, Eulderink F, Westesson PL (1986). Osteoarthritis and internal derangement of the temporomandibular joint: a light microscopic study. J. Oral Maxillofac. Surg..

[7] Wang XD, Kou XX, Mao JJ, Gan YH, Zhou YH (2012). Sustained inflammation induces degeneration of the temporomandibular joint. J. Dent. Res..

[8] Wang XD (2014). Deterioration of mechanical properties of discs in chronically inflamed TMJ. J. Dent. Res..

[9] Abe S (2013). Stress analysis in human temporomandibular joint affected by anterior disc displacement during prolonged clenching. J. Oral Rehabil..

[10] Iwasaki LR (2009). Temporomandibular joint loads in subjects with and without disc displacement. Orthop. Rev. (Pavia).

[11] Coombs MC (2017). Structure–function relationships of temporomandibular retrodiscal tissue. J. Dent. Res..

[12] Tanaka E, van Eijden T (2003). Biomechanical behavior of the temporomandibular joint disc. Crit. Rev. Oral Biol. Med..

[13] Kou XX (2011). 17β-estradiol aggravates temporomandibular joint inflammation through the NF-κB pathway in ovariectomized rats. Arthritis Rheum..

[14] Xue XT (2017). Progesterone attenuates temporomandibular joint inflammation through inhibition of NF-κB pathway in ovariectomized rats. Sci. Rep..

[15] Király K (1997). Specimen preparation and quantification of collagen birefringence in unstained sections of articular cartilage using image analysis and polarizing light microscopy. Histochem. J..

[16] Scapino RP, Obrez A, Greising D (2006). Organization and function of the collagen fiber system in the human temporomandibular joint disk and its attachments. Cells Tissues Organs.

[17] Puchtler H, Waldrop FS, Valentine LS (1973). Polarization microscopic studies of connective tissue stained with picro-Sirius red FBA. Beitr. Pathol..

[18] Liu Y (2013). Hierarchical intrafibrillar nanocarbonated apatite assembly improves the nanomechanics and cytocompatibility of mineralized collagen. Adv. Funct. Mater..

[19] Dokukin ME, Sokolov I (2012). Quantitative mapping of the elastic modulus of soft materials with HarmoniX and PeakForce QNM AFM modes. Langmuir.

[20] West PA, Torzilli PA, Chen C, Lin P, Camacho NP (2005). Fourier transform infrared imaging spectroscopy analysis of collagenase-induced cartilage degradation. J. Biomed. Opt..

[21] West PA, Bostrom MP, Torzilli PA, Camacho NP (2004). Fourier transform infrared spectral analysis of degenerative cartilage: an infrared fiber optic probe and imaging study. Appl. Spectrosc..

[22] Missirlis D (2011). Effect of the peptide secondary structure on the peptide amphiphile supramolecular structure and interactions. Langmuir.

[23] Mlyniec A, Mazur L, Tomaszewski KA, Uhl T (2015). Viscoelasticity and failure of collagen nanofibrils: 3D coarse-grained simulation studies. Soft Mater..

[24] Andriotis OG (2015). Structure-mechanics relationships of collagen fibrils in the osteogenesis imperfecta mouse model. J. R. Soc. Interface.

[25] Flint MH, Craig AS, Reilly HC, Gillard GC, Parry DA (1984). Collagen fibril diameters and glycosaminoglycan content of skins--indices of tissue maturity and function. Connect. Tissue Res..

[26] Ahn HJ (2007). Age-related changes in the microarchitecture of collagen fibrils in the articular disc of the rat temporomandibular joint. Arch. Histol. Cytol..

[27] Wen CY (2012). Collagen fibril stiffening in osteoarthritic cartilage of human beings revealed by atomic force microscopy. Osteoarthr. Cartil..

[28] Lee GJ (2013). Nanostructural and nanomechanical responses of collagen fibrils in the collagenase-induced Achilles tendinitis rat model. J. Nanosci. Nanotechnol..

[29] Nia HT (2015). High-bandwidth AFM-based rheology is a sensitive indicator of early cartilage aggrecan degradation relevant to mouse models of osteoarthritis. J. Biomech..

[30] Chan SM, Neu CP, Duraine G, Komvopoulos K, Reddi AH (2010). Atomic force microscope investigation of the boundary-lubricant layer in articular cartilage. Osteoarthr. Cartil..

[31] Bi X, Yang X, Bostrom MP, Camacho NP (2006). Fourier transform infrared imaging spectroscopy investigations in the pathogenesis and repair of cartilage. Biochim. Biophys. Acta.

[32] Xia Y, Mittelstaedt D, Ramakrishnan N, Szarko M, Bidthanapally A (2011). Depth-dependent anisotropies of amides and sugar in perpendicular and parallel sections of articular cartilage by Fourier transform infrared imaging. Microsc. Res. Technol..

[33] Boskey A, Pleshko Camacho N (2007). FT-IR imaging of native and tissue-engineered bone and cartilage. Biomaterials.

[34] Jackson M, Choo LP, Watson PH, Halliday WC, Mantsch HH (1995). Beware of connective tissue proteins: assignment and implications of collagen absorptions in infrared spectra of human tissues. Biochim. Biophys. Acta.

[35] van Meurs JB (1999). Increased vulnerability of postarthritic cartilage to a second arthritic insult: accelerated MMP activity in a flare up of arthritis. Ann. Rheum. Dis..

[36] Manka SW (2012). Structural insights into triple-helical collagen cleavage by matrix metalloproteinase 1. Proc. Natl. Acad. Sci. USA.

[37] Asakawa-Tanne Y (2015). Effects of enzymatic degradation after loading in temporomandibular joint. J. Dent. Res..

[38] Yuya PA, Amborn EK, Beatty MW, Turner JA (2010). Evaluating anisotropic properties in the porcine temporomandibular joint disc using nanoindentation. Ann. Biomed. Eng..

[39] Baldwin SJ, Quigley AS, Clegg C, Kreplak L (2014). Nanomechanical mapping of hydrated rat tail tendon collagen I fibrils. Biophys. J..

[40] Broder C (2013). Metalloproteases meprin and meprin are C- and N-procollagen proteinases important for collagen assembly and tensile strength. Proc. Natl. Acad. Sci. USA.

[41] Bekhouche M (2016). Determination of the substrate repertoire of ADAMTS2, 3, and 14 significantly broadens their functions and identifies extracellular matrix organization and TGF-β signaling as primary targets. FASEB J..

[42] Kwok J (2014). Atomic force microscopy reveals age-dependent changes in nanomechanical properties of the extracellular matrix of native human menisci: implications for joint degeneration and osteoarthritis. Nanomedicine.

[43] Suzuki Y (2013). Visualization of structural changes accompanying activation of *N*-methyl-d-aspartate (NMDA) receptors using fast-scan atomic force microscopy imaging. J. Biol. Chem..

[44] Wang XD (2012). Progression of cartilage degradation, bone resorption and pain in rat temporomandibular joint osteoarthritis induced by injection of iodoacetate. PLoS ONE.

